# An Innovative Compact System to Measure Skiing Ground Reaction Forces and Flexural Angles of Alpine and Touring Ski Boots

**DOI:** 10.3390/s23020836

**Published:** 2023-01-11

**Authors:** Giuseppe Zullo, Pierluigi Cibin, Lorenzo Bortolan, Michele Botteon, Nicola Petrone

**Affiliations:** 1 Department of Industrial Engineering, University of Padua, Via Venezia 1, 35131 Padova, Italy; 2 CeRiSM, Sport Mountain and Health Research Centre, University of Verona, 38068 Rovereto, Italy; 3 Department of Neurosciences, Biomedicine and Movement Sciences, University of Verona, 37124 Verona, Italy; 4 Tecnica Group, 31040 Giavera del Montello, Italy

**Keywords:** ski touring, alpine ski, ski boot, ground reaction forces, snow sports

## Abstract

Skiing is a popular winter activity spanning various subdisciplines. Key hardware are ski boots, bindings, and skis, which are designed to withstand loads generated during skiing. Obtaining service forces and moments has always been challenging to researchers in the past. The goal of the present study is to develop and test a lightweight and compact measurement system to obtain the Ground Reaction Forces and the kinematics for ski touring and alpine ski. To do so, we adapted two six-axis load cells to fit into ski touring and alpine skis adding 20 mm height and 500 g weight to the original ski. To measure kinematics, we created custom angular sensors from rotary potentiometers. The system was tested indoors using a force platform and motion capture system before a first set of field tests in which the sensors were used to measure ski touring and alpine skis kinetics and kinematics. Validation trials showed maximum errors of 10% for kinetics and 5% for kinematics. Field tests showed data in agreement with previous findings on the topic. The results of this study show the possibility of using our system to study biomechanics and equipment performances for ski touring, alpine skiing, and possibly other disciplines.

## 1. Introduction

Skiing is a popular winter activity that is practiced each year by 400 million people [1]. Several subdisciplines had developed with different techniques and gear requirements. The most established is alpine skiing, in which the skier descends ski slopes with a pair of ski boots attached to skis. Ski bindings provide solid connection between the two during skiing and allow automatic release upon reaching hazardous loads. Another widespread skiing activity is ski touring or backcountry skiing. Key difference with respect to alpine ski is the fact that the skier does not rely on ski lifts to gain altitude but uses special gear to climb the mountain. More in detail, seal skins glued under the skis prevent the skis from backsliding during uphill motion, and special boots and bindings allow better mobility of the ankle and the relative motion between heel and ski during the ascending phase.

Besides its popularity, skiing is also quite dangerous, especially to one’s lower limbs. To address this problem, 2-mode release bindings were introduced. These bindings release the ski from the boot either when torque between heel and tip of the boot or the forward leaning force on the heel exceed threshold values. Thanks to the adoption of these bindings, a dramatically reduction in lower leg fractures was observed [2]. More advanced bindings with more than 2 release modes exist [3]. However, knee injuries still remain the most common issue in skiing [4,5,6]. Indeed, advances in the equipment are aiming to reduce the risks associated with skiing as well as testing procedures to evaluate binding effectiveness [7], which add to the ISO 9462 testing protocol [8]. However, to improve advances on this field, a proper knowledge of the Ground Reaction Forces (GRF) generated during skiing is needed.

Despite the general agreement on this point, the actual measurement of the six components GRF during skiing, without disturbing the skier, is still an open issue. Indeed, obtaining such measurements while keeping a minimal modification of the gear size and weight is a complex task.

Hull and Mote were among the first to address the problem of measuring the full GRF with custom six-axis load cells placed between the ski and binding [9]. Their system allowed to measure the forces under both front and rear binding; however, it required a custom ski and added about 2 kg of weight per ski.

After the introduction of carving skis, the attention to loads generated at the ski-binding interface lead to the development of several dynamometric systems with different approaches: Scott and Yoneyama [10] developed calibrated force plates interposed between the ski and the bindings, whereas Senner et al. [11] developed a dynamometric plate to be interposed between the ski binding and the ski boot.

Later then, Klous et al. made use of customized force platforms based on piezoelectric multiaxial load cells to obtain the forces and of cameras to obtain kinematics, following an approach adopted in several previous tests. The system was quite accurate but still added 7 kg to the skier [12].

More recently, Meyer et al. built instrumented plates to be mounted between the ski boot and binding [13]. The main pro of their system is that no customization is required for the gear; however, some load components as well as the distribution of loads between front and rear binding is unavailable. This approach was also used by Petrone et al. [14] when measuring the loads acting at the ski–snow interface of an elite paralympic skier by means of two customized low profile force plates, one under each binding.

Other authors focused more on the ski boot. Petrone et al. did some experiments to investigate the stiffness of ski boots on torsional and flexural stresses, pairing indoor and field tests with instrumented ski boots and measuring components of the GRF and kinematics [15,16,17]. Zullo et al. instrumented a ski touring boot to measure torsion loads during an outdoor climb and ski [18].

To our knowledge, no study reported a compact and lightweight system to measure both GRF and kinematics over a wide range of skiing disciplines and gear, such as ski touring and alpine ski. To address this lack in the current literature, we developed a measurement system to obtain GRF and kinematics for ski touring. Since most works were focused on alpine skiing rather than ski touring, where mass is critical, the main goal is to develop a light and compact system while maintaining the full GRF components and adding kinematics. The novel system also aims to be easily installable to several pair of skis and bindings and to give information about the distribution of loads between front and rear bindings, not only being limited to measuring the whole GRF under the ski. This design choice could allow one to better understand how the load is transferred between the boot and the ski.

## 2. Materials and Methods

### 2.1. Kinetic Sensor

A pair of M3564F six-axis load cells (Sunrise Instruments, Shanghai, China) were used to measure the loads between skis and ski boots. Geometrically, each load cell is shaped like a steel cylinder with a diameter of 65 mm, height of 10 mm and a weight of 0.190 kg. The three components of forces and moments are measured with reference to the cylinder axis (Z) and two radial orthogonal axes (X, Y). The selected load transducer has full scale force of 2500 N in X, Y axes and 5000 N in Z axis, full scale moment of and 200 Nm in X, Y axes and 100 Nm in Z axis. Nonlinearity of the load cell is 0.33% and hysteresis 0.38% of the full scale for each direction. Maximum crosstalk is of 2.26% of the full scale. The manufacturer provided a calibration matrix to obtain the six load components from the six physical channels of the load cell.

More in detail, the two load cells were secured to custom made aluminum plates to interface with the ski and the bindings. The bottom plates, connecting the load cells to the skis, match screw pattern of alpine ski bindings to allow their connection either to skis or to bindings plates. Top plates, connecting loadcells to the ski bindings, were instead designed differently for touring and alpine ski, to match the different hole pattern of the bindings. For the study, Alpinist 13 (Marker, Straubing, Germany) ski touring bindings and XComp 20 (Marker, Straubing, Germany) alpine ski bindings were used.

**Figure 1:**
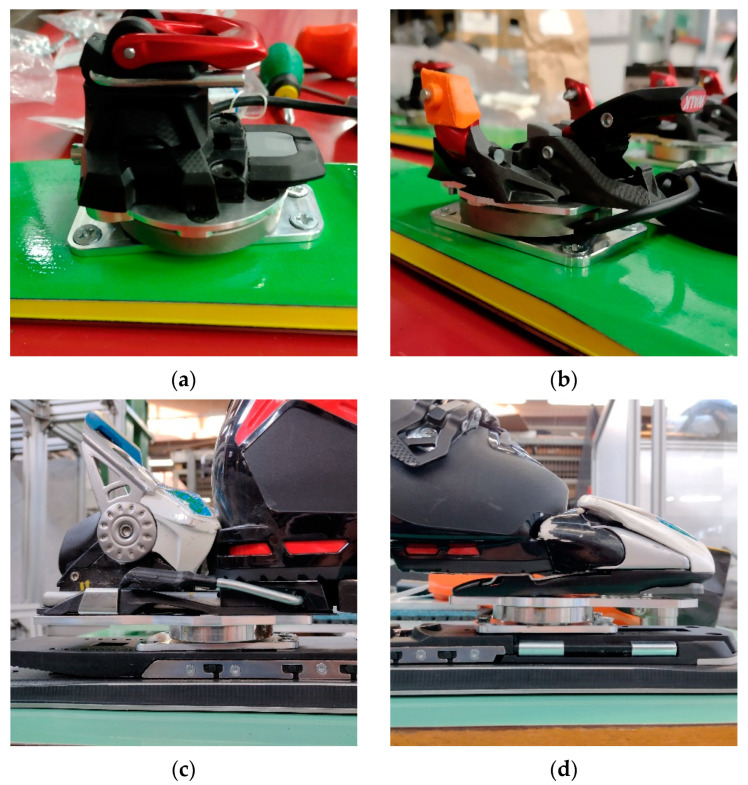
Load cells and plates connecting skis to ski bindings: (**a**) ski touring, rear piece; (**b**) ski touring, front piece; (**c**) alpine ski, rear piece; (**d**) alpine ski, front piece.

The load cell system resulted in an added height between ski and boot of 20 mm, with respect to the original system. Total weight added to the system is of 0.5 kg/ski. Since the measurement system was implemented only on the right ski, we added two steel cylinders to the left ski in substitution to the load cell to have a symmetric system. Figure 1 shows the assembly of the dynamometric system for ski touring and alpine ski.

### 2.2. Kinematic Sensors

Kinematics of the ski touring boot were obtained using a set of SV01 Series rotary potentiometers (muRata, Nagaokakyo, Japan). For the alpine ski boot, a set of electrogoniometers (Biometrics, Ladysmith, VA, USA) was also used based on the solutions implemented in previous works [16,17]. These sensors provide a validated measure of joint angles without requiring a hinge joint between the segments [19].

#### 2.2.1. Ski Touring

A ZeroG Tour Pro ski touring boot (size: 26.5 MP; weight: 1.32 kg; Tecnica, Italy) was instrumented for testing the sensor system. Angles were measured using rotary potentiometers mounted on custom cases on the ski boot. The cases were manufactured using a MEGA 3D printer (Anycubic, Shenzhen, China) with a PLA filament at 100% infill density.

The angle between ski and shell (φ_SS_) was measured on medial and lateral side in the correspondence of the ski touring boot binding insert. The sensor was positioned inside a custom 3D printed case and mounted on the ski touring boot. The sensor measured the angle with respect to a fixed shaft glued to the ski touring binding (see Figure 1b). To allow the boot to detach from the binding without damaging the sensor, the case is designed to have a flexible beam to separate the sensor from the shaft before opening the ski binding.

**Figure 2:**
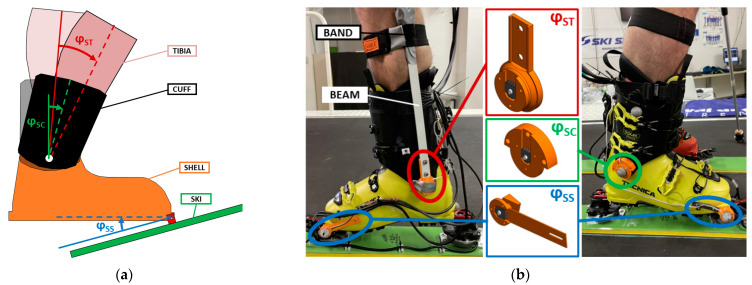
Ski touring boot kinematic sensors: (**a**) kinematic chain of the boot, zero is set with the unloaded ski boot (solid lines); (**b**) sensor application to the medial and lateral side of the boot.

To measure the other angles, we took advantage of the design of the boot. Indeed, a pair of aluminum bushes is fixed between shell and cuff to obtain a hinge joint approximately located near the medial and lateral malleoli. Exploiting this feature, we screwed a shaft to each bush to have a shell reference for the rotary potentiometers. On the lateral side, the shaft connects to a rotary potentiometer fixed to the cuff to measure shell/cuff (φ_SC_) angle. On the medial side, the potentiometer was connected to a beam hinged to the shaft (fixed to the shell) and connected on the other ending to the tibia with a soft band to get shell/tibia (φ_ST_) angle. The CAD rendering of the cases for the sensors and their application to the ski boot are shown in Figure 2.

#### 2.2.2. Alpine Ski

**Figure 3:**
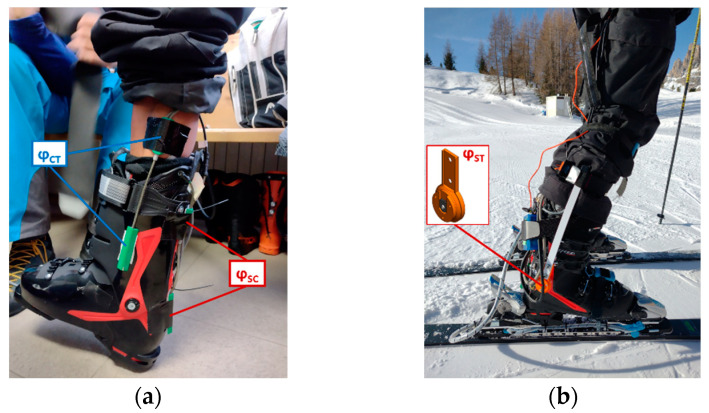
Alpine ski boot kinematic sensors: (**a**) medial view of the alpine ski boot with the electrogoniometric sensors; (**b**) lateral view of the alpine ski boot with the force sensors under the binding and the potentiometric sensor φ_ST_ in the lateral side.

A Speed Machine 130 (size: 26.5 MP; weight: 1.5 kg; Nordica, Italy) was instrumented for testing the sensor system. Angle between shell and cuff (φ_SC_) was measured using an electrogoniometer positioned on the back of the ski boot. The same sensor was also used to measure the angle between cuff and tibia (φ_CT_) by positioning the sensor on the medial side. Finally, the sensor used to measure φ_ST_ in ski touring was adapted to the new boot to obtain the angle between shell and tibia at the lateral side. The sensors applied to the alpine ski boot are visible in Figure 3.

### 2.3. System Validation

Before performing the outdoor test, we validated the system by comparing the output from instrumented skis and ski boots to a SMART DX-7000 motion capture system (BTS, Milan, Italy) paired with a P-6000 force platform (BTS, Milan, Italy).

**Figure 4:**
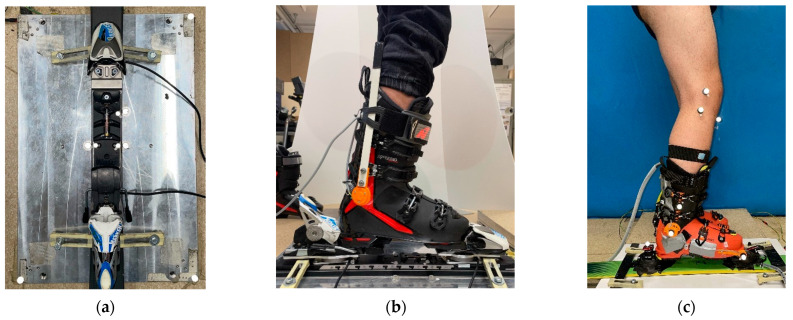
System validation setup: (**a**) instrumented ski mounted on the force platform, top view; (**b**) instrumented alpine ski mounted on the force platform during the test, lateral view; (**c**) ski touring boot with markers for angular sensors validation.

To perform the validation test, the ski was fixed to the force platform and the position of the load cells with respect to the ski origin and the platform reference system was measured by applying reflective markers in critical locations. Then, a volunteer subject performed: side and vertical loading, internal/external turning, forward/backward leaning, and twisting. The resulting loads and moments on each axis were synchronously recorded by the ski load cells and the force platform and compared between the two systems. To validate the angles, we positioned reflective markers on the ski, ski boot, and leg of the subject, and we compared the angles obtained with the potentiometers to the one reconstructed with the motion capture. Figure 4 shows the setup of the system for the kinetic and kinematic validation test.

### 2.4. Field Tests

Field tests were conducted in December 2021 at Col Gallina ski area (Belluno, Italy) to validate the system in the field both for ski touring and alpine ski. Ski touring tests were conducted both on groomed (on-piste) and fresh (off-piste) snow. A volunteer experienced ski instructor (weight: 82 kg, height: 180 cm) wore the instrumented ski touring boots paired with instrumented ZeroG 95 skis (Blizzard, Mittersill, Austria). The measurement system was tested during uphill climbing and downhill skiing both on fresh and groomed snow.

After the completion of ski touring tests, the subject performed some descents on a groomed slope wearing the instrumented alpine ski boot and the load cell system mounted on a pair of Dobermann Spitfire 70 PRO skis (Nordica, Italy).

Data of load cells and kinematic sensors were synchronously collected using a SoMat eDAQ lite (HBM, Darmstadt, Germany) fitted in a backpack at 1 kHz sampling rate.

### 2.5. Data Analysis

**Figure 5:**
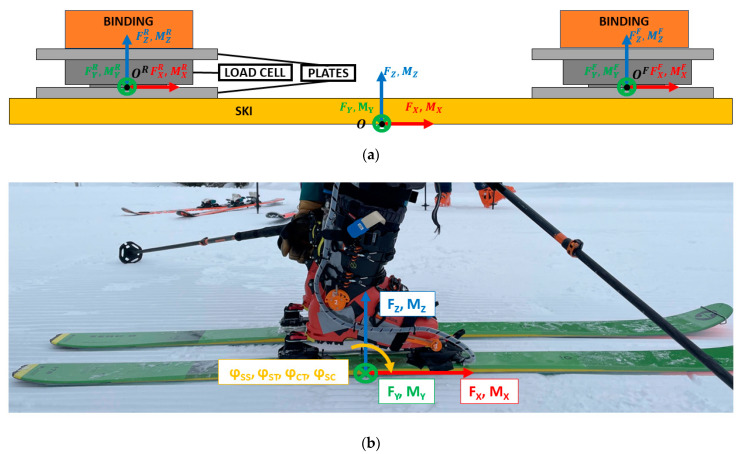
(**a**) Load cells position with respect to ski; (**b**) assembled system with GRF and kinematics reference systems and sign conventions.

Data of the test were analyzed using MATLAB (MathWorks, Natick, MA, USA). First, data from the two six-axis load cells were subjected to a coordinate transformation and combined to obtain the resultant GRF with respect to the ski origin (ski center mark, on the bottom surface of the ski), according to the reference system shown in Figure 5 and Equations (1) and (2). In these following equations, **F**, **M** are the vectors of forces and moments, and **OO^R^** and **OO^F^** are the vectors connecting the origin of the ski (**O**) to the origins of the front (**O^F^**) and rear (**O^R^**) load cells. Force is obtained by adding the forces of the two loadcells (Equation (1)), moment is obtained by addition of the moments on each loadcell plus the cross product between force and its lever arm with respect to ski origin (Equation (2)).

**F** = **F**^F^ + **F**^R^, (1)

**M** = **M**^F^ + **M**^R^+ **OO^F^** × **F**^F^+ **OO^R^** × **F**^R^, (2)

Forces and kinematics were low-pass filtered using a fourth-order Butterworth zero-phase filter with a cutoff frequency of 5 Hz [9].

For ski touring uphill trials, we identified the gait cycle based on the rising edge of the vertical force of the rear load cell (i.e., heel strike). In downhill, cycles were identified on the change of sign of turning moment from the negative to the positive sign. (i.e., beginning of a right turn).

## 3. Results

Validation trial results are reported in Table 1. During the test, maximum errors were found in the F_Y_ and M_Z_ components of the GRF. Conveniently, on the components of major interest (F_Z_, M_X_, M_Y_), the error with respect to the force platform was equal or lower than 10%. Kinematics showed good results with less than 5% of error with respect to the motion capture system.

**Figure 6:**
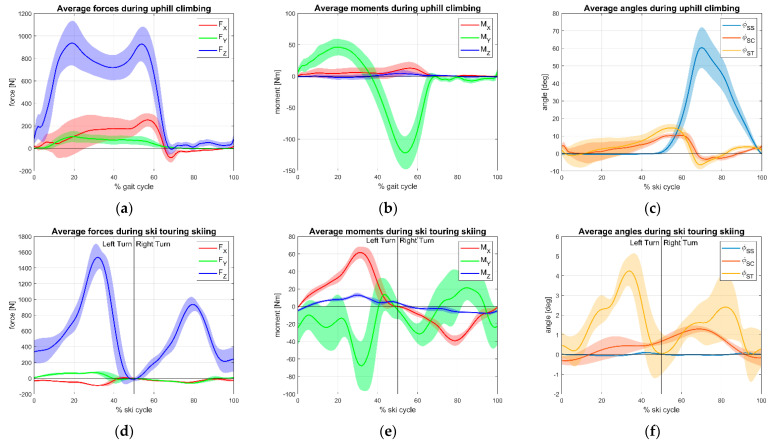
Ground reaction forces and kinematics during ski-touring session: (**a**) uphill forces; (**b**) uphill moments; (**c**) uphill angles; (**d**) downhill forces; (**e**) downhill moments; (**f**) downhill angles.

**Figure 7:**
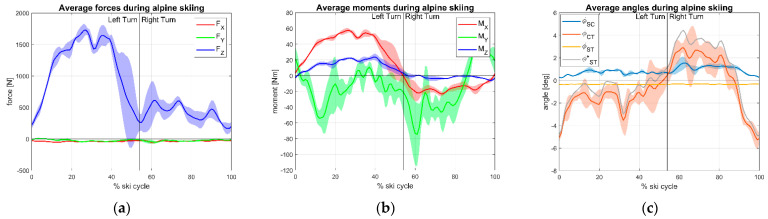
Ground reaction forces and kinematics during alpine ski session: (**a**) forces; (**b**) moments; (**c**) angles.

Regarding the field test, normalized cycles both for ascent and descend phase of ski touring are represented in Figure 6 for ski touring and Figure 7 for alpine ski. Table 2 reports the min, max, and mean values observed in the average normalized cycle for each discipline.

Maximum values of Z component of the GRF were obtained during skiing, with average peaks of 1539 N and 1820 N for touring and alpine, respectively.

In ski touring ascent, timing between signals shows that tibia (φ_ST_) and cuff (φ_CT_) increase together until the cuff contacts the shell and the tibia angle tends to increase, due to liner compliance; then, after the heel has started lifting from the ski (φ_SS_) to complete the step, the two signals decrease together with again a larger negative peak of the tibia due to the posterior liner and calf muscles compliance. Sagittal moment (M_Y_) clearly indicates the weight shifting from the rear to the front of the ski center.

In downhill trials, as expected, left turns (in which the instrumented right ski is on the outer side) loads are greater as shown by F_Z_ and M_X_ magnitude. Together with M_Y,_ these three components showed the highest GRF magnitude values. These components, indeed, are related to skiing technique. F_Z_ and M_X_ relate to the magnitude and angle of incidence of pressure on the skis during the turn and the distribution of weight between the internal and external leg. M_Y_ is related to the posture of the skier (forward/backward leaning) indicating where the weight is placed by the skier longitudinally to the ski.

## 4. Discussion

Results of the preliminary tests showed data consistent with our expectations. The load cell system was able to return GRF both in ski touring and alpine skiing. Comparison of collected data with past works showed agreement of forces and moments with previous studies showing slightly higher values [12,13,20]. Maximum values reached suggest that the system could be implemented in other disciplines with higher loads (e.g., involving jumps) since the signals were still far from the nominal total full scale of the load cells. Alternatively, load cells could be downsized to further reduce the weight of the system (e.g., by manufacturing them in aluminum). Plates used to connect the sensors to skis and bindings were robust and practical to assemble and disassemble. The reconstruction of GRF from the individual load cells was accurate compared to the motion capture system (at least for load components of higher interest) and can resolve the load components in different reference points of the system, such as the boot hinges.

Some work should still be done to improve the system on the sensor side to reduce the errors in some of the components of the GRF. Further lab tests will aim to investigate the origin of these errors and provide strategies to reduce them, such as correction factor matrices for the possible coupling error between Moment and Force resultant channels. A more compact and lightweight data acquisition system could be used to gather data from load cells and angle sensors, reducing the disturbance to the skier.

About kinematics, the embedded system that we developed could be helpful to deepen the knowledge on ski touring biomechanics also in the real field, without the space limitations of an external system (such as marker-based motion capture) to obtain the relative angles of the lower limb. The rotary potentiometers on the boot were proved a to be viable solution to provide ski touring angles since they are cost-effective, quite accurate, and proved reliable even in the harsh field conditions in which they were tested. For the touring boot, the sensors integrated quite good with the joints of the boot with front binding and between its shell and cuff, exploiting the construction characteristics of the boot. However, the set of sensors could be improved for alpine skiing with higher resolution sensors given the low rotations which were measured during the test. In particular, the null output of the potentiometric φ_ST_ signal was explained by the large constructive misalignment of the alpine boot lateral hinge that, given the small values of the tibia movement relative to the shell, were masked by the flexural compliance of the sensing beam strapped to the tibia. This inconvenient was solved by estimating angle φ_ST_ from the series addition of φ_SC_ and φ_CT_ as reported in Table 2.

Given the results of field tests, we believe that our system could result as an innovation to existing solutions in the range of possibilities offered to study GRF in skiing. Its peculiarity is also the possibility of obtaining the GRF separately for anterior and posterior bindings, obtaining more detailed boundary conditions to the design of gear (e.g., ski boots and binding release mechanics). This characteristics was found only in a previous work, which, however, added 7 times more weight to the ski with respect to ours [12]. Moreover, differently to other systems, we added the possibility of measuring ski boot kinematics and the transferability of the load cell system to several skis with no modifications on the existing gear but the mounting of bindings on aluminum plates. Indeed, to transfer the load cells to different skis, only some basic hardware and less than one hour of work are required, making our tool also useful to companies that need to test several skis in a single day.

Future research will be focused on collecting data with the presented system on several athletes and gear. This data could be used to investigate skiing biomechanics toward an improvement of performance and safety.

## Figures and Tables

**Table 1:** Peak values of resultant GRF during system validation.

	Forces [N]			Moments [Nm]			Angles [deg]		
	F_X_	F_Y_	F_Z_	M_X_	M_Y_	M_Z_	φ_ST_	φ_CT_	φ_SS_
Force Platform	88.3	98.4	836.9	52.4	68.7	11.5	11.8	6.6	43.7
Instrumented Ski	82.0	74.4	877.3	47.1	69.8	9.3	12.1	6.3	41.8
Error [%]	7.1	24.4	4.8	10.1	1.6	18.8	2.8	4.6	4.4

**Table 2:** Min, max, and mean values obtained during the tests.

Test		Forces [N]			Moments [Nm]			Angles [deg]			
		F_X_	F_Y_	F_Z_	M_X_	M_Y_	M_Z_	φ_SS_	φ_SC_	φ_CT_	φ_ST_
Ski touring, ascent	min	−87.6	11.9	−40.5	0.1	−124.0	−0.1	−0.4	−3.2	-	−6.5
	max	248.0	119.8	906.0	13.1	44.1	5.9	60.5	10.4	-	14.7
	mean	75.8	56.7	432.1	4.5	−13.2	2.2	14.4	2.8	-	3.7
Ski touring, descent	min	−117.0	−58.3	−10.2	−38.9	−76.3	−7.7	−0.1	−0.3	-	0.0
	max	−19.9	77.7	1539.1	61.9	12.8	13.3	0.1	1.3	-	4.2
	mean	−61.6	11.1	567.0	5.6	−21.5	1.3	0.0	0.5	-	1.5
Alpine ski, descent	min	−15.8	−49.0	255.8	−23.1	−63.8	−10.8	-	0.2	−5.3	−4.9 *
	max	18.4	12.1	1820.7	57.4	60.2	19.1	-	1.5	2.9	4.5 *
	mean	4.2	−19.1	897.0	13.5	−4.2	2.1	-	0.8	−0.7	0.2 *

* φ_ST_ is obtained by adding φ_CT_ and φ_CT_ as applied in [17].

## Data Availability

Not applicable.
